# Web Portals in Primary Care: An Evaluation of Patient Readiness and Willingness to Pay for Online Services

**DOI:** 10.2196/jmir.8.4.e26

**Published:** 2006-10-26

**Authors:** Kenneth G Adler

**Affiliations:** ^1^Department of Family and Community MedicineUniversity of ArizonaTucsonAZUSA

**Keywords:** Internet, communication, primary health care, electronic mail, patient access to records, Web-based services, payment schemes

## Abstract

**Background:**

Online Web communication between physician and patient has been proposed by leading primary care organizations as a way to enhance physician-patient communication, but lack of payment for this service has acted as a significant barrier to implementation.

**Objective:**

This study evaluates current patient readiness and willingness to pay for online services in a fairly typical urban family medicine practice.

**Methods:**

All patients that visited the author for medical care during a one-month period in the spring of 2006 were anonymously surveyed with a one-page survey instrument that inquired about demographics, willingness to pay a small annual fee for online services, the greatest fee they were willing to pay, and their most desired service.

**Results:**

A total of 346 patients out of 2380 active patients in the study practice (14.5%) were surveyed. The valid survey response rate was 95.1% (329/346.) Three quarters, or 75.4%, of patients had Internet access. The group with the highest access were 18- to 29-year-olds (97%), and the group with the least access were those 70 years and up (56%) (*P* < .001). Categorized by employment, students and employed patients had the best access at 92% and 87%, respectively, and retirees and disabled patients had the worst access at 66% and 42%, respectively (*P* < .001). Of all patients with Internet access, 74.6% (n = 185) were willing to pay a small annual fee for one or more of the following online services: viewing of parts of their medical record, messaging with their physician, medication refills, appointment requests, and billing inquiries. Willingness to pay did not vary significantly by age (*P* = .06). Of all respondents, regardless of Internet access, 47.1% (n = 155) were willing to pay US $10 or more per year, with the median amount being US $20. Of those with Internet access (n = 248), 60.1% (n = 149) were willing to pay US $10 or more per year, and 31% were willing to pay US $50 or more per year. The three most important services to patients with Internet access (n = 248), in order of importance, were emailing with their physician (34%), Internet viewing of their medical record (22%), and medication refills (11%) (*P* < .001).

**Conclusions:**

This study suggests that patients of all ages are currently ready and willing to pay a small annual fee for online services with their primary care physician’s office. If 47.1% of a practice of 2500 patients each paid US $10 per year for online services, the annual revenue generated would be US $11775. Not only does this study support the economic feasibility of patient Web portals, but it suggests that online services could form a new line of revenue for primary care physicians.

## Introduction

We live in a time when online communication has become commonplace in numerous service industries, yet to date that has not been the case in health care – at least as far as doctor-patient communication goes. Based on a paucity of research, there is a perception that patients want online communication with their physicians and their offices, but aren’t willing to pay for it. A Harris Interactive survey of over 2000 online adults in 2002 showed that almost all (90%) respondents would like to communicate with their physicians online. This same survey showed that only 37% were willing to pay for it [1]. A prior Harris Interactive survey had shown that online adults are often unhappy with their ability to communicate with their physician and his/her office, and the majority felt that online access would improve communication [2]. A randomized controlled trial by Lin et al showed that patients in an academic internal medicine practice who used a Web portal had higher satisfaction with physician-patient communication than those who did not [3]. The American Academy of Family Physicians, the American College of Physicians, and the Society of General Internal Medicine have all proposed online communication as one tool to help revitalize primary care in the United States and to help improve doctor-patient communication and patient access [4-6].

Despite this generally acknowledged desire to implement widespread physician-patient online communication, a number of barriers exist, including lack of reimbursement, concerns about patient privacy and confidentiality, medicolegal concerns, practical workflow concerns, and physician fears of being overwhelmed by online messages [7].

Physicians fear that they will be inundated with online patient messages, and they voice frustration with not being reimbursed for these services [8]. Yet several studies of early adopters have not found physicians to be overwhelmed by patient emails [9-12]. The development of confidential Web portals that are linked to an electronic health record (EHR) have addressed many of the privacy, confidentiality, and workflow concerns, but lack of reimbursement remains a major obstacle. In one survey of physician users of Web messaging, 80% of physicians said they would be more willing to engage in online patient communication if it was reimbursed [12]. A few insurance companies have started paying physicians for direct online communication between doctor and patient for care of a clinical problem (e-consult) at a rate of about US $25 to $30, or half that of an intermediate office visit, but they remain the exception [13-15].

Patient Web portals represent a significant advance beyond traditional physician-patient email. They are online sites that can be free-standing or integrated with an EHR. Often they are tied to a personal health record (PHR), or, in the most recent definition of PHRs, Web portals are actually synonymous with PHRs as long as they include the ability to record and update key aspects of a person’s health history, like their medication list, allergies, and health problem list. These sites allow patients to securely message their physician and to request medication refills and/or appointments. In some cases, when linked to an EHR, they allow patients to view and/or download some components of their medical record, such as medication and problem lists [16-18]. They offer convenient asynchronous communication for patient and physician alike [9,10]. Increased use of Web portals can reduce physician phone traffic and increase practice efficiency [19].

Despite all their advantages and the expressed desire of patients and primary care physician organizations to utilize Web portals, a number of questions about reimbursement remain. Do patients want online access to their physician office enough to pay a small annual fee for it, perhaps in addition to an e-consult transaction fee? Such a subscription fee might more than defray the direct and indirect costs of offering these services. It is axiomatic that a person’s willingness to pay for services indicates a higher level of true demand than their simply indicating they would use the service if it were free.

This study was undertaken to determine the true level of demand for online services in a fairly typical family medicine practice in Tucson, Arizona and to answer the following related questions: What are the demographics of the patients most interested and least interested in these services, and what is their current Internet connectivity? Are patients willing to pay a nominal annual fee for these services and, if so, how much? Which of these services do patients value the most?

## Methods

### Practice Site

The practice used was the author’s. This practice is part of a family medicine office that has 3 physicians and a nurse practitioner. Patients have a clearly identified physician and rarely, if ever, see one of the physician partners. The nurse practitioner primarily assists each physician with acute care patient visits and patient coverage when a physician is out of the office. In other words, the study practice, although part of a group practice, functions like a solo practice with a one-third time nurse practitioner. The office uses an EHR and has offered free “one-way” email to patients since 2000. If patients sign an email agreement, they are permitted to email the practice messages and the practice responds by phone. This service has never been heavily utilized. Based on EHR data, the practice size is 2380 active patients, defined as patients seen by the author in the last 36 months who are still alive. This practice sees virtually all insurances available in the community, including self-pay and Medicaid (called AHCCCS in Arizona.) It includes the full range of socioeconomic groups and ages (newborn to over 100 years). Being an urban family practice that does not involve obstetrics, it is likely more heavily weighted toward geriatric patients than those in a smaller or rural community. As a mature practice, it is largely closed to new patients except for family members of current patients.

### Survey

The author’s patients were given a one-page survey entitled “Web Portal Survey” (Multimedia Appendix) when they arrived for an appointment with the author from April 10, 2006 to May 11, 2006. A receptionist handed out the survey. The survey did not have any specific patient identifiers other than gender. Age and employment were only generally identified by broad categories. Surveys were collected by reception when the patients checked out. They were given to the author in a nonsequential manner at the end of each day. The author did not discuss the content of the self-explanatory surveys with the patients other than to occasionally inquire if they had completed one. Patients were only allowed to complete one survey. If they returned for a follow-up visit during the study period (as determined by reviewing the schedule), they did not get another survey to complete. Parents completed surveys on behalf of patients younger than 18 years, and caregivers occasionally completed surveys for elderly or disabled patients who were unable to complete them themselves.

The one-page survey consisted of strictly check box answers and was easy to complete. The questions asked about simple demographics, information on Internet access, and willingness to pay a “small fee” for any of five different Web portal services. Quantification of the fee was obtained by inquiring the maximum annual fee respondents were willing to pay, and this was followed by asking which of the five services were most important to the respondent.

Given the manner in which the data were collected, handled, and entered into the database, the data were, by deliberate design, de-identified. This was done to honor the statement at the top of the survey that this was an anonymous survey, thus encouraging patients to be frank in their responses. The study was deemed to be exempt by the Human Research Committee (Institutional Review Board) of Tucson Medical Center.

### Analysis

After collecting the surveys, the data were entered nonsequentially into an Access database (Microsoft, USA) and analyzed using select queries. When comparing respondent groups for significant differences, contingency tables were created and the StatsDirect statistical software v2.5.7 (Chesire, UK) was used. Analysis was done with the Fisher exact test. *P* values less than .05 were considered significant.

## Results

### Return Rate

During the study period, 346 unique patients were seen. Of those, 337 completed and returned a survey (return rate of 97.4%). A total of 8 surveys were invalidated due to missing data (not answering whether they had Internet access and/or not making any responses to the question on willingness to pay a small fee for one or more of the five online services listed on the survey). Thus, the number of valid surveys was 329 (response rate of 95.1%).

### Internet Access

In the study practice, 75.4% of patients had Internet access. Internet access varied significantly by age, gender and employment (Table 1). Specifically, in terms of age, 18- to 29-year-olds had the highest access (97%), and patients 70 years and older had the least access (56%) (*P* < .001; Fisher exact test, 8x2 contingency table). Of note, 41% of the respondents were 60 and older.Students and employed patients had the best access, 92% and 87%, respectively, and retirees and disabled patients had the worst access, 66% and 42%, respectively (*P* < .001; Fisher exact test, 6x2 contingency table). Males were more likely to have Internet access than females (*P* = .02; Fisher exact test, 3x2 contingency table).

**Table 1:** Demographics and Internet access

	**All Patients(N = 329)**	**Patients With Internet(N = 248)**
	**n (%)**	**n (%)**
**Gender**		
Male	135 (41)	111 (45)
Female	192 (58)	136 (55)
Unknown	2 (1)	1 (0)
**Age**		
Under 18	14 (4)	12 (5)
18–29	29 (9)	28 (11)
30–39	26 (8)	21 (8)
40–49	50 (15)	44 (18)
50–59	63 (19)	47 (19)
60–69	61 (19)	46 (19)
70 and up	72 (22)	40 (16)
Unknown	14 (4)	10 (4)
**Employment**		
Student	26 (8)	24 (10)
Employed	142 (43)	123 (50)
Unemployed	12 (4)	8 (3)
Disabled	24 (7)	10 (4)
Retired	123 (37)	81 (33)
Unknown	2 (1)	2 (0)
**Internet access**		
Yes	248 (75.4)	
No	81 (24.6)	

### Annual Fee

Of all patients with Internet access, 74.6% (n = 185) were willing to pay a small annual fee for one or more of the following online services: emailing with their physician, medication refills, viewing parts of their medical record, appointment requests, and billing inquiries. On a per service basis, 67% of patients with Internet access were willing to pay a “small fee” for “secure email” with their physician, 62% for online refills, 60% to review their medical record, 57% to request appointments, and 52% to make billing inquiries (Table 2). The differences in these responses were significant (*P* = .04; Fisher exact test, yes vs no vs no response 5x3 contingency table).

**Table 2:** Willingness of patients with Internet access to pay a small fee for specific online services (N = 248)

**Service**	**Yesn (%)**	**Non (%)**	**No Responsen (%)**
Email with doctor	165 (67)	76 (31)	7 (3)
Medication refills	153 (62)	79 (32)	16 (6)
Viewing record	148 (60)	89 (36)	11 (4)
Appointment request	141 (57)	93 (37)	14 (6)
Billing inquiry	128 (52)	101 (41)	19 (8)

The majority of patients with Internet access in all age ranges were willing to pay a small fee for at least one of the five online or Web portal services (Table 3). Although this willingness to pay ranged from 60% for those in their 50s to 90% for those in their 30s, these differences were not statistically significant (*P* = 0.06; Fisher exact test, yes vs no response 8x2 contingency table).

**Table 3:** Willingness of patients with Internet access willingness to pay for at least one of the five services, by age (N = 248)

**Age (years)**	**n**	**Yes**	**%**
Less than 18	12	9	75
18–29	28	17	61
30–39	21	19	90
40–49	44	36	82
50–59	47	28	60
60–69	46	35	76
70 and older	40	33	83
Unknown	10	8	80
**All**	248	185	75

Of all respondents (N = 329), regardless of Internet access, 47.1% (n = 155) were willing to pay US $10 or more per year, with a median amount of US $20 (Table 4). Of those with Internet access (n = 248), 60.1% (n = 149) were willing to pay US $10 or more per year, and 31% (n = 46) were willing to pay US $50 or more per year. Of those who were disabled (n = 24), 29% were willing to pay US $10 or more per year.

**Table 4:** Maximum fee patients were willing to pay for online services (N = 329)

**Amount (US $)**	0	< 10	10	20	50	100	> 100
**n**	140	34	41	68	35	10	1

### Online Services

As Table 5 shows, the three most important services to patients with Internet access (n = 248), in order, were emailing with their physician (34%), viewing their record online (22%), and medication refills (11%) (*P* < .001; Fisher exact test, most important vs not most important 7x2 contingency table). Note that 12% of those without Internet access (10/81) were still willing to pay for the service.

**Table 5:** Most important online service and willingness to pay

**Most Important Service**	**All Patients(N = 329)**	**Patients With Internet(N = 248)**
	**n %**	**n %**
Email	93 (28)	84 (34)
Record viewing	57 (17)	55 (22)
Medication refills	31 (9)	28 (11)
Multiple selections	21 (6)	18 (7)
Appointment requests	15 (5)	15 (6)
Billing information	0 (0)	0 (0)
No response	112 (34)	48 (19)
**Willingness to pay for at least one service**	**195 (59)**	**185 (75)**

## Discussion

In the study practice, three quarters of adults are online and three quarters of those stated they are willing to pay a small annual fee for at least one of the five listed Web portal services. Even 12% of non-Internet users are willing to pay (presumably using the service through a friend or relative’s Internet access or a public source like the library). Willingness to pay for Web portal services did not appear to vary significantly by age for those who already have Internet access. Nearly half of all patients and 60% of patients with Internet access were willing to pay at least US $10 per year for one or more of these services. Over 30% of patients with Internet access were willing to pay US $50 or more per year.

This study showed that no single online service stood out as the obvious favorite, but patient-physician email generated the highest level of interest, followed by online viewing of personal medical records, and online medication refills. No one chose billing inquiries as the service they valued the most. These findings are consistent with a February 2005 Harris Poll in which 80% of respondents indicated an interest in asking online questions of their physician, 69% in making online appointments, 69% in receiving test results online, and 67% in online medication renewal [20].

Like the 2002 Harris study that showed only 37% of online adults would be willing to pay for email with their physician [1], the February 2005 Harris Poll also reported that only 36% of online adults were willing to pay to send and receive emails from their doctor [20]. That stands in marked distinction to the finding here of 67% of patients being willing to pay for email with their physician. It is unknown whether the 2638 adults in the more recent Harris study had regular primary care physicians. It is conceivable that many did not and that people who have an established primary care physician relationship are more willing to pay for these services. Still another estimate of some patient willingness to pay for online patient-physician correspondence comes from an academic internal medicine practice in Colorado. In their study, 48% of all patients (both Web portal and non-portal users) were willing to pay for electronic correspondence with their physician. However, the amount they were willing to pay was small, at a mean of US $4 and median of US $2 per message [3].

A small annual fee could add up quickly if it could be collected. Ideally, it would be collected electronically at the time a user signs up and then annually thereafter. Just a US $10 per year fee for a practice of 2500 patients with 47.1% willingness to pay would translate into US $11775 per year of additional revenue for the involved physician. Web portals are relatively inexpensive and the current cost of one commercial product is about US $900 per physician per year [21]. A question this study did not address was whether patients are willing to pay both a small annual subscription fee and a per-transaction fee for e-messaging. This issue deserves further study.

A limitation of this study is that even though the top of the survey form stated “This is a completely confidential study,” and even though the author avoided discussing the survey with them, patients may have been more inclined to give answers they thought he wanted to hear because they were in the office for care that day. This theoretically could have biased the results toward more favorable responses regarding payment.

Although the author’s practice is felt to be fairly diverse and to adequately reflect the demographics of Tucson, no two practices are alike. If this practice is representative of Tucson, Arizona, can its findings be generalized to other communities? The author believes so, but it would be wise to establish this with similar studies conducted elsewhere. Evidence that the practice is representative lies in the Internet access statistic of 75.4%, which is consistent with a 2006 phone poll that reported 77% of US adults are now online [22]. And even if this study is representative of family medicine populations, would the findings for internal medicine practices differ significantly? Given the results of Table 3, which show 83% of patients over age 70 willing to pay for at least one of the five online services, a rate similar to other age groups, it appears likely that these results can be generalized to internal medicine as well.

A concern raised by this study is that one vulnerable, higher need population, the disabled, had relatively low Internet access (42%), and of those who had access, only 29% were willing to pay US $10 or more for online services. Since it is logical to think that people with medical disabilities would benefit more than most others by engaging in online services, the likely explanation for both the lower access and the lower “interest” is financial constraint – having to spend limited income on other needs. It would be useful to learn how interested disabled patients would be if these services were free for them.

What people say and what they do are not always the same. It would be most revealing to implement the services mentioned here with an annual fee of say, perhaps, US $15 and an email fee US $25 per message used to manage a clinical concern and see how many patients sign up for each.

## Abbreviations

EHR: electronic health record
PHR: personal health record

## Appendix

### Web Portal Survey

This is a completely confidential survey.

**Patient’s age:** □ under 18 □ 18–29 □ 30–39 □ 40–49 □ 50–59 □ 60–69 □ 70 and older

**Patient’s sex:** □ male □ female

**Patient’s job:** □ student □ employed □ unemployed □ disabled □ retired

**Do you have Internet access?** □ Yes □ No

**Would you be willing to pay a small fee for the following services?**

- Secure Internet viewing of your medical record □ Yes □ No
  (includes: Health summary, medication list, problem list, allergy list, lab results)
- Secure two way email with Dr. Adler □ Yes □ No
- Secure requests for medication refills (via Internet) □ Yes □ No
- Secure requests for appointments (via Internet) □ Yes □ No
- Secure email for billing questions / issues □ Yes □ No

**If you answered yes to any of the above services, what is the MOST that you would be willing to pay per year?**

□ less than $10 □ $10 □ $20 □ $50 □ $100 □ more than $100

**Please check the one service that would be MOST important to you:**

(check only one)

□ Secure Internet viewing of your medical record

□ Secure two way email with Dr. Adler

□ Secure requests for medication refills (via Internet)

□ Secure requests for appointments (via Internet)

□ Secure email for billing questions / issues

**COMMENTS:**

## Multimedia Appendix

Presentation of Study Findings	 
	
