# Supplementary material for: Web Portals in Primary Care: An Evaluation of Patient Readiness and Willingness to Pay for Online Services
Source: J Med Internet Res. 2006 Oct 26;8(4):e26. doi: 10.2196/jmir.8.4.e26 (PMC1794005; doi:10.2196/jmir.8.4.e26)
Supplement: Supplementary file 1 [file jmir_v8i4e26_app1.ppt]

## Slide 1
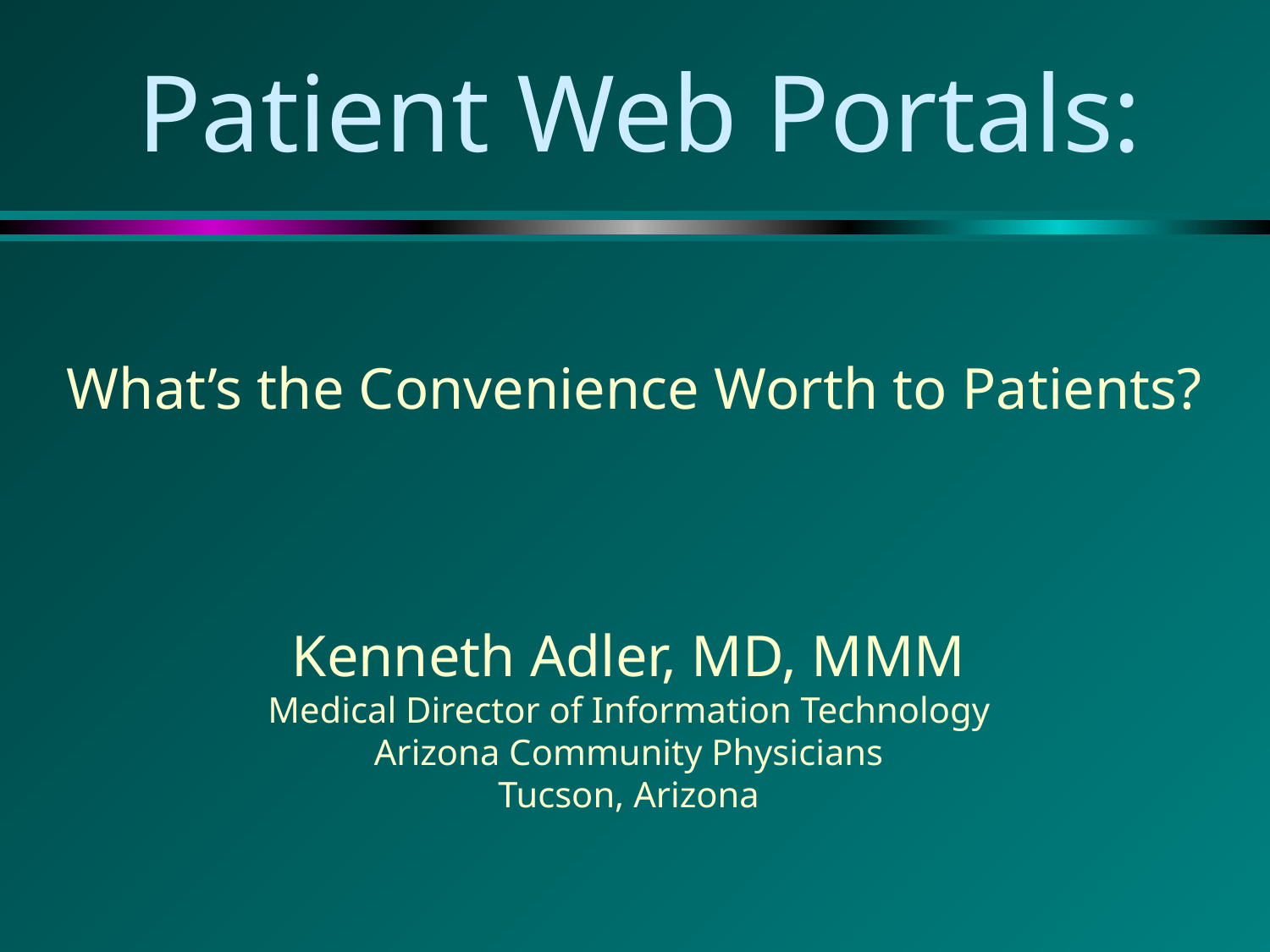

# Patient Web Portals:
What’s the Convenience Worth to Patients?
Kenneth Adler, MD, MMM
Medical Director of Information Technology
Arizona Community Physicians
Tucson, Arizona

## Slide 2
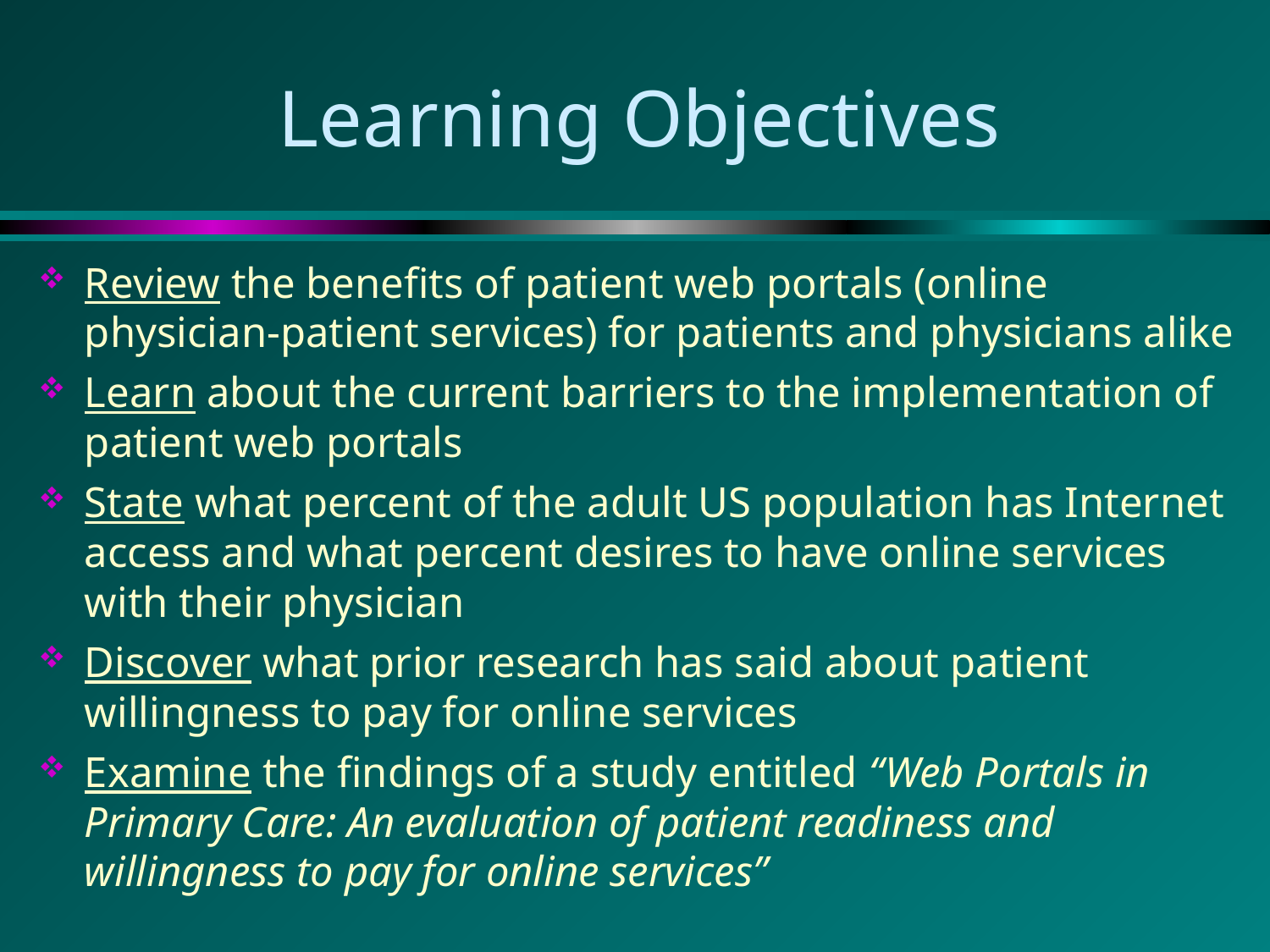

# Learning Objectives
Review the benefits of patient web portals (online physician-patient services) for patients and physicians alike
Learn about the current barriers to the implementation of patient web portals
State what percent of the adult US population has Internet access and what percent desires to have online services with their physician
Discover what prior research has said about patient willingness to pay for online services
Examine the findings of a study entitled “Web Portals in Primary Care: An evaluation of patient readiness and willingness to pay for online services”

## Slide 3
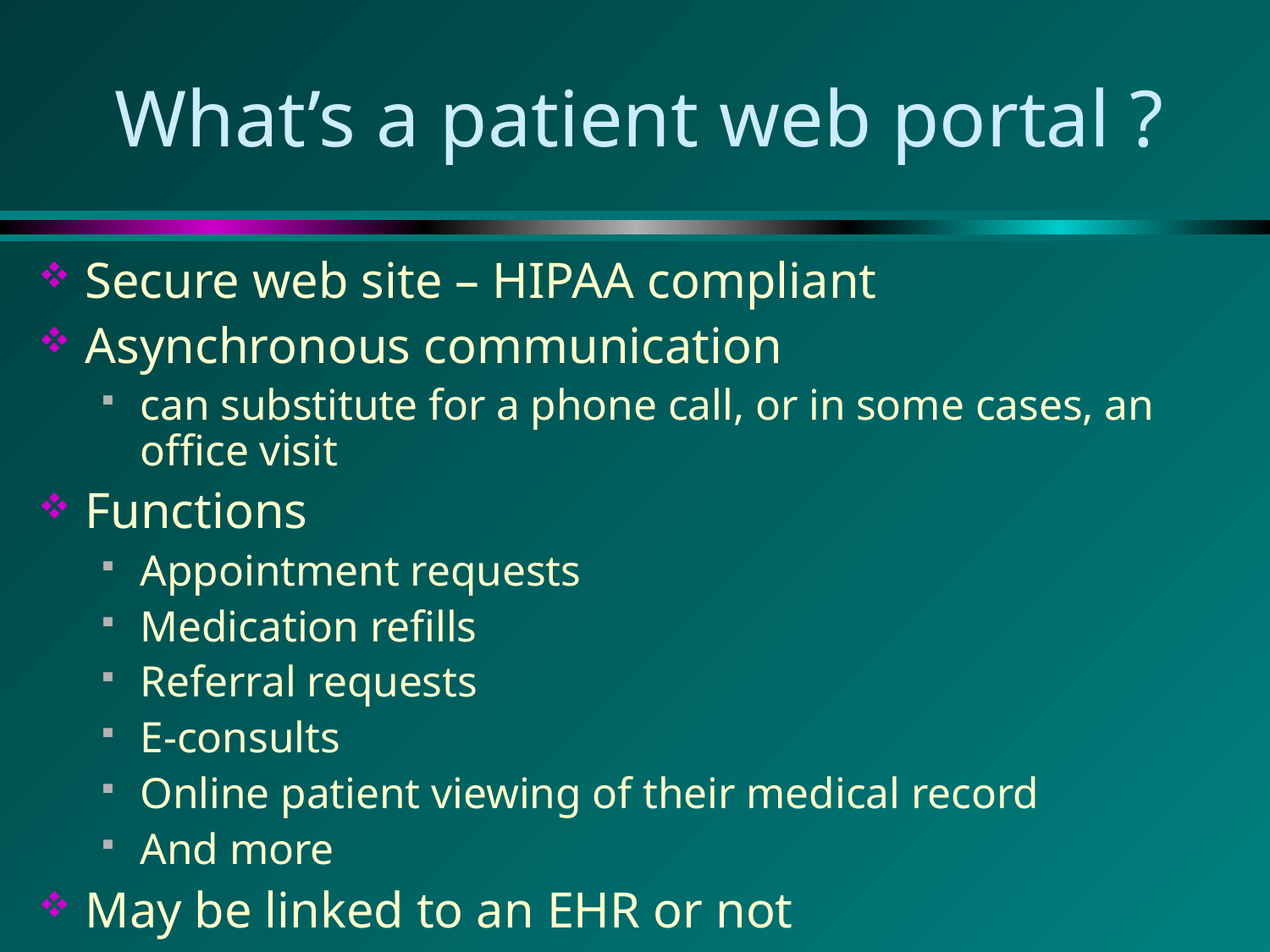

# What’s a patient web portal ?
Secure web site – HIPAA compliant
Asynchronous communication
can substitute for a phone call, or in some cases, an office visit
Functions
Appointment requests
Medication refills
Referral requests
E-consults
Online patient viewing of their medical record
And more
May be linked to an EHR or not

## Slide 4
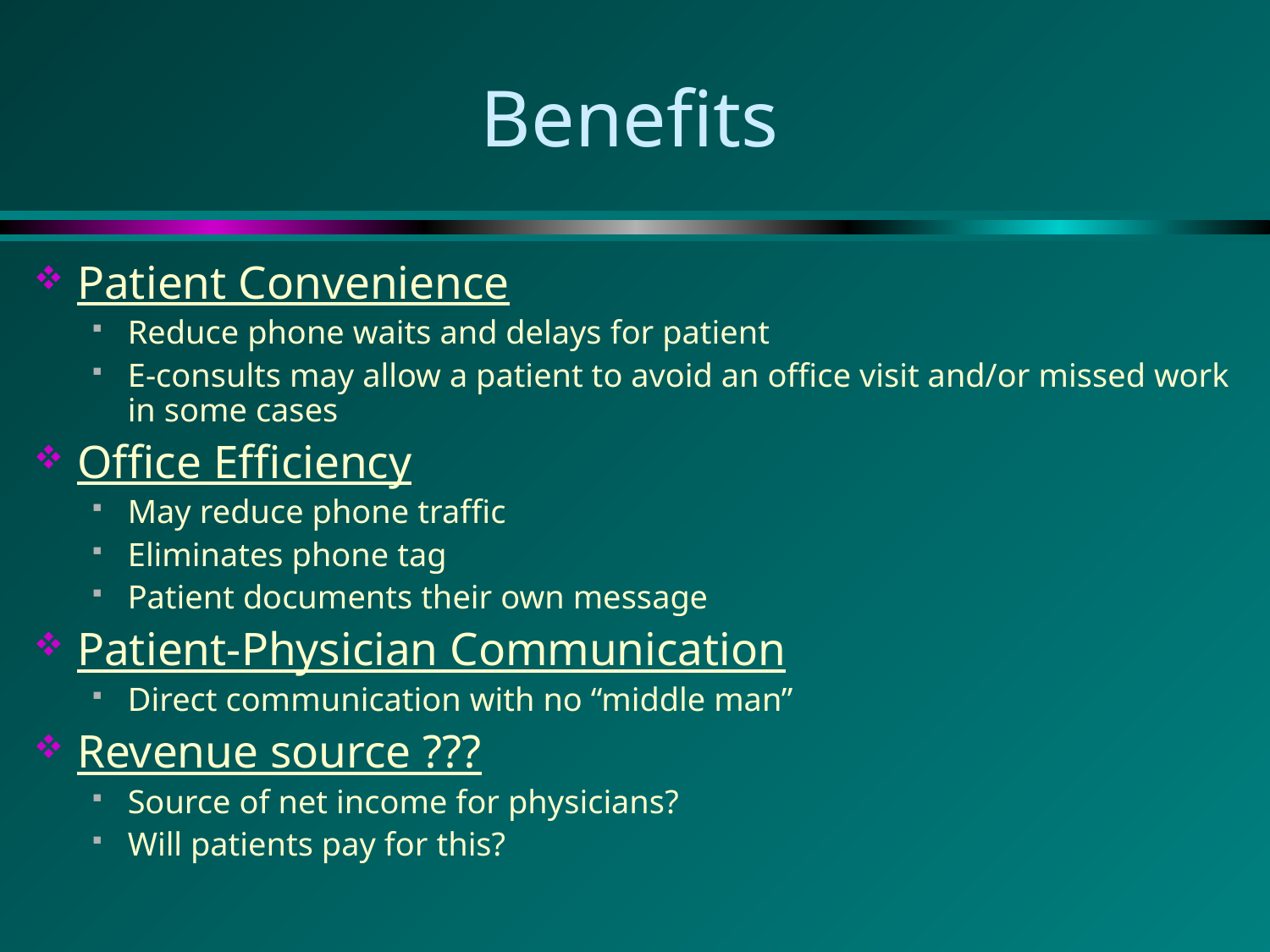

# Benefits
Patient Convenience
Reduce phone waits and delays for patient
E-consults may allow a patient to avoid an office visit and/or missed work in some cases
Office Efficiency
May reduce phone traffic
Eliminates phone tag
Patient documents their own message
Patient-Physician Communication
Direct communication with no “middle man”
Revenue source ???
Source of net income for physicians?
Will patients pay for this?

## Slide 5
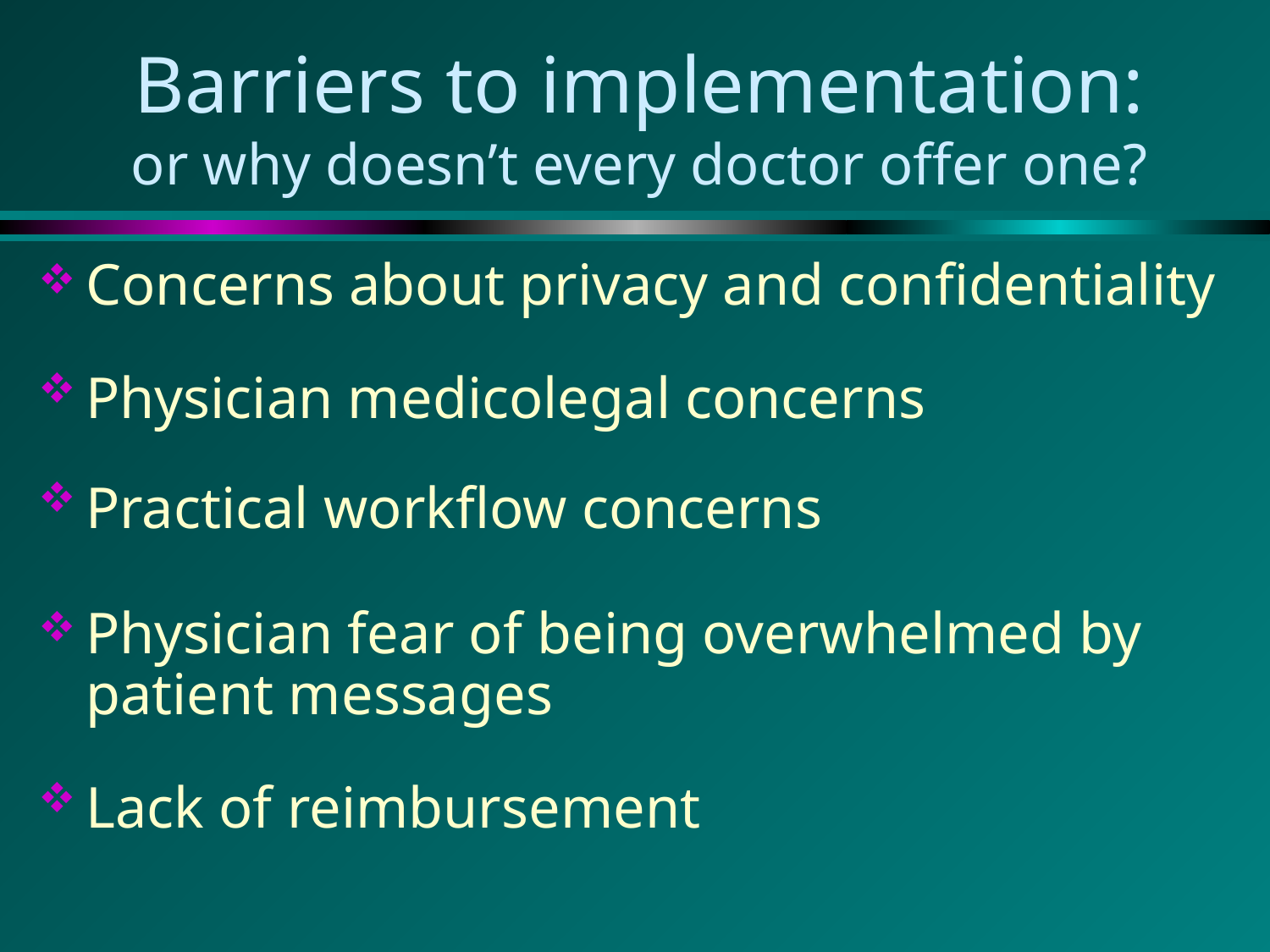

# Barriers to implementation: or why doesn’t every doctor offer one?
Concerns about privacy and confidentiality
Physician medicolegal concerns
Practical workflow concerns
Physician fear of being overwhelmed by patient messages
Lack of reimbursement

## Slide 6
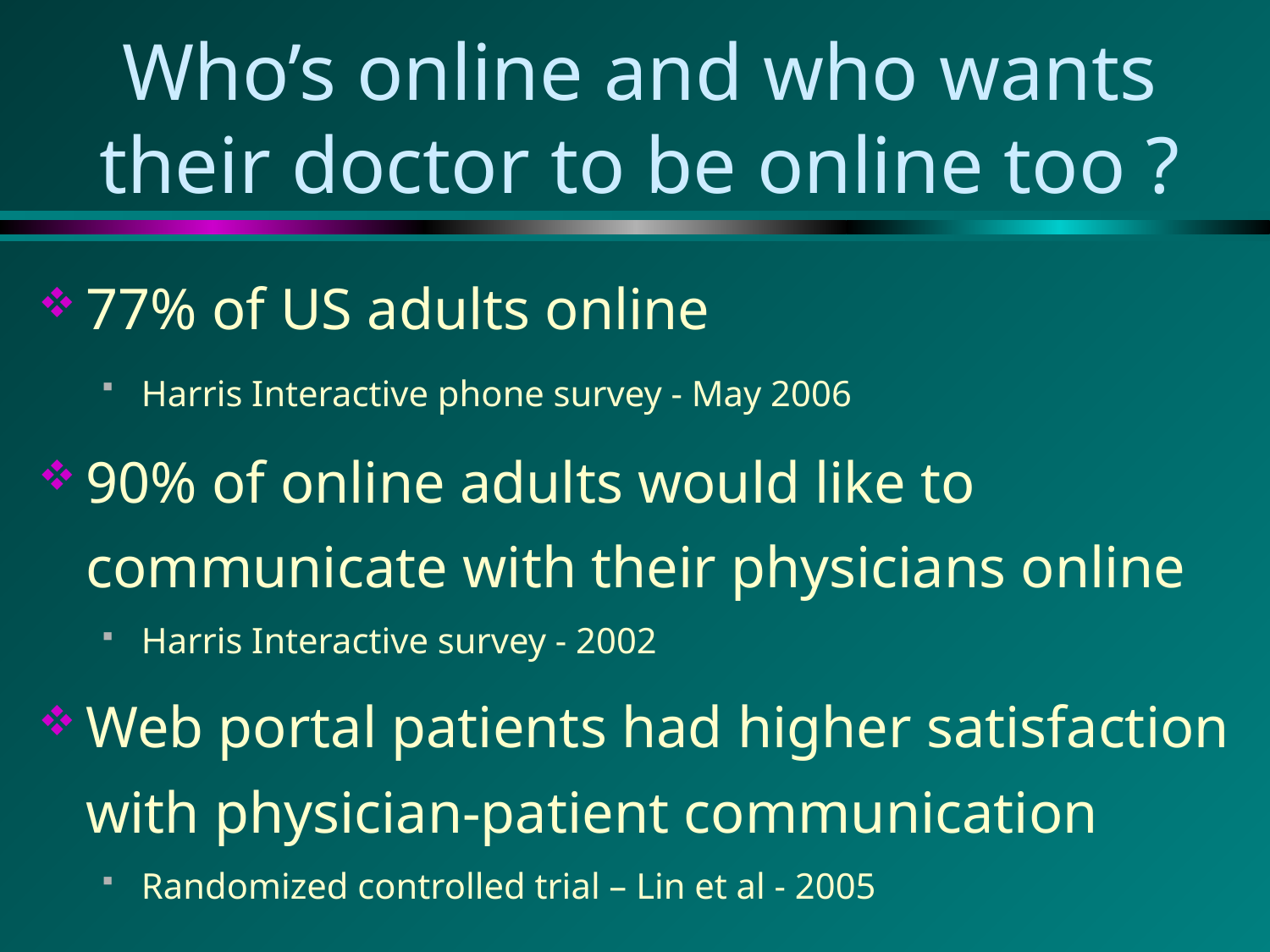

# Who’s online and who wants their doctor to be online too ?
77% of US adults online
Harris Interactive phone survey - May 2006
90% of online adults would like to communicate with their physicians online
Harris Interactive survey - 2002
Web portal patients had higher satisfaction with physician-patient communication
Randomized controlled trial – Lin et al - 2005

## Slide 7
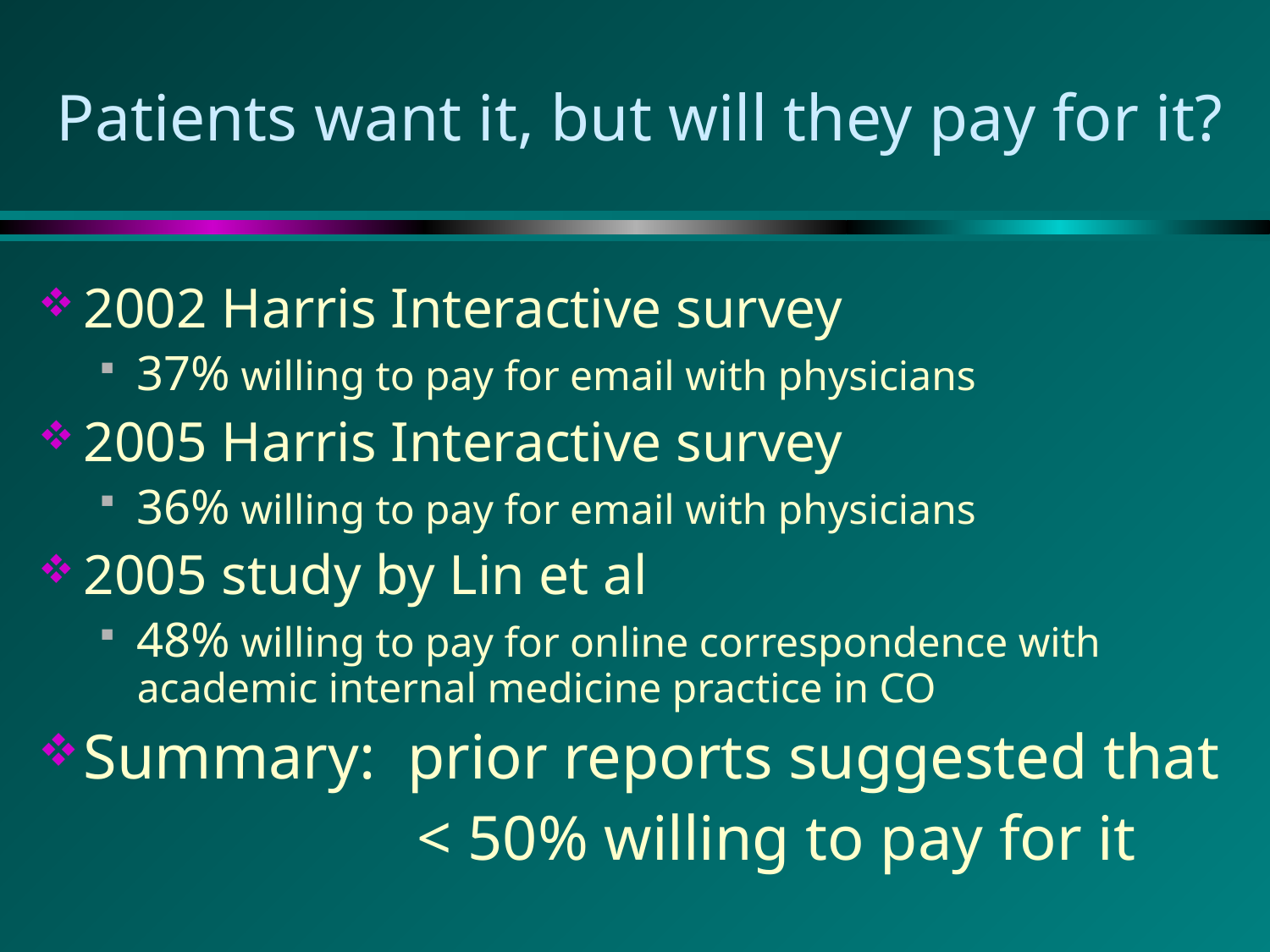

# Patients want it, but will they pay for it?
2002 Harris Interactive survey
37% willing to pay for email with physicians
2005 Harris Interactive survey
36% willing to pay for email with physicians
2005 study by Lin et al
48% willing to pay for online correspondence with academic internal medicine practice in CO
Summary: prior reports suggested that
 < 50% willing to pay for it

## Slide 8
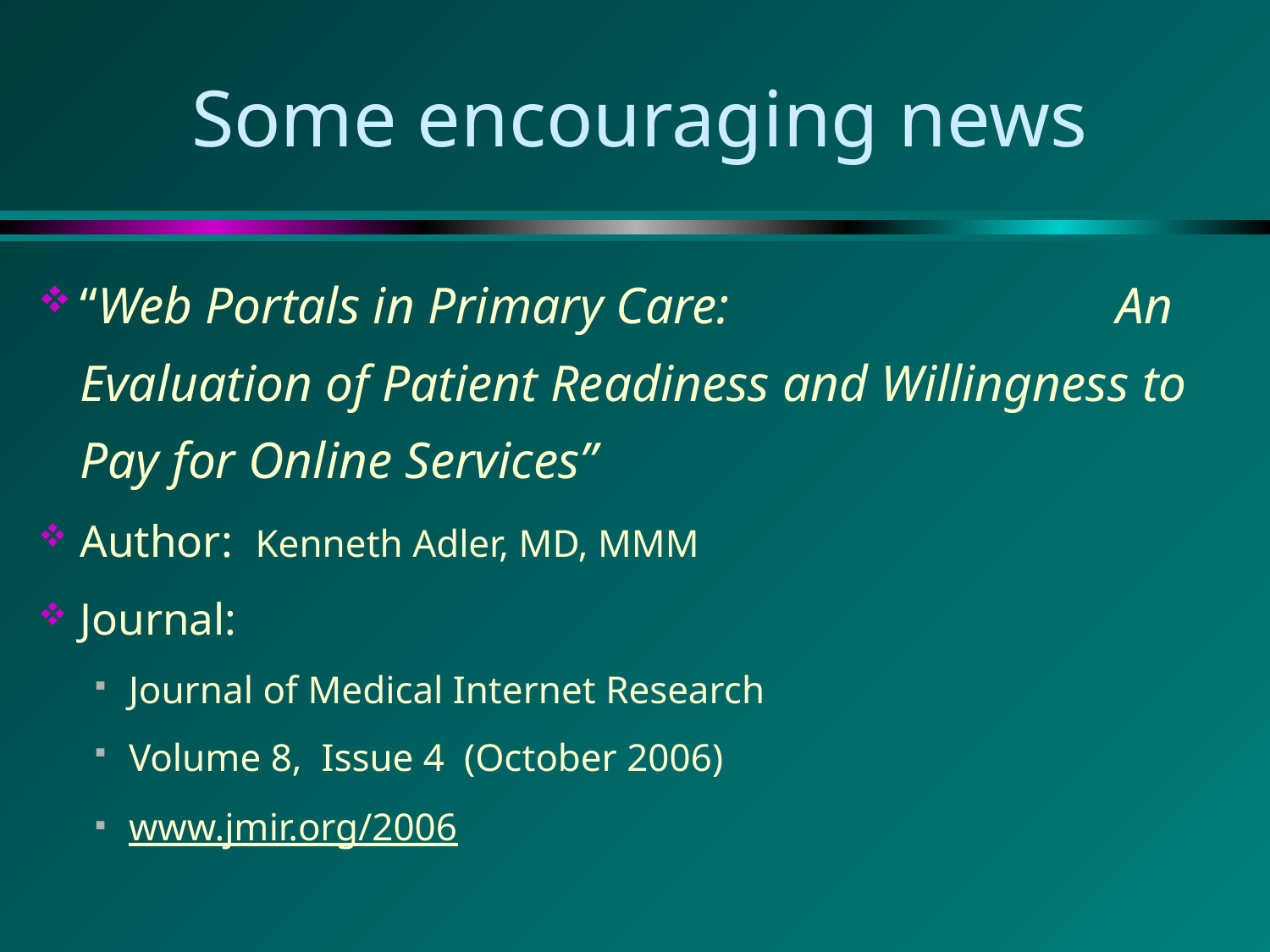

# Some encouraging news
“Web Portals in Primary Care: An Evaluation of Patient Readiness and Willingness to Pay for Online Services”
Author: Kenneth Adler, MD, MMM
Journal:
Journal of Medical Internet Research
Volume 8, Issue 4 (October 2006)
www.jmir.org/2006

## Slide 9
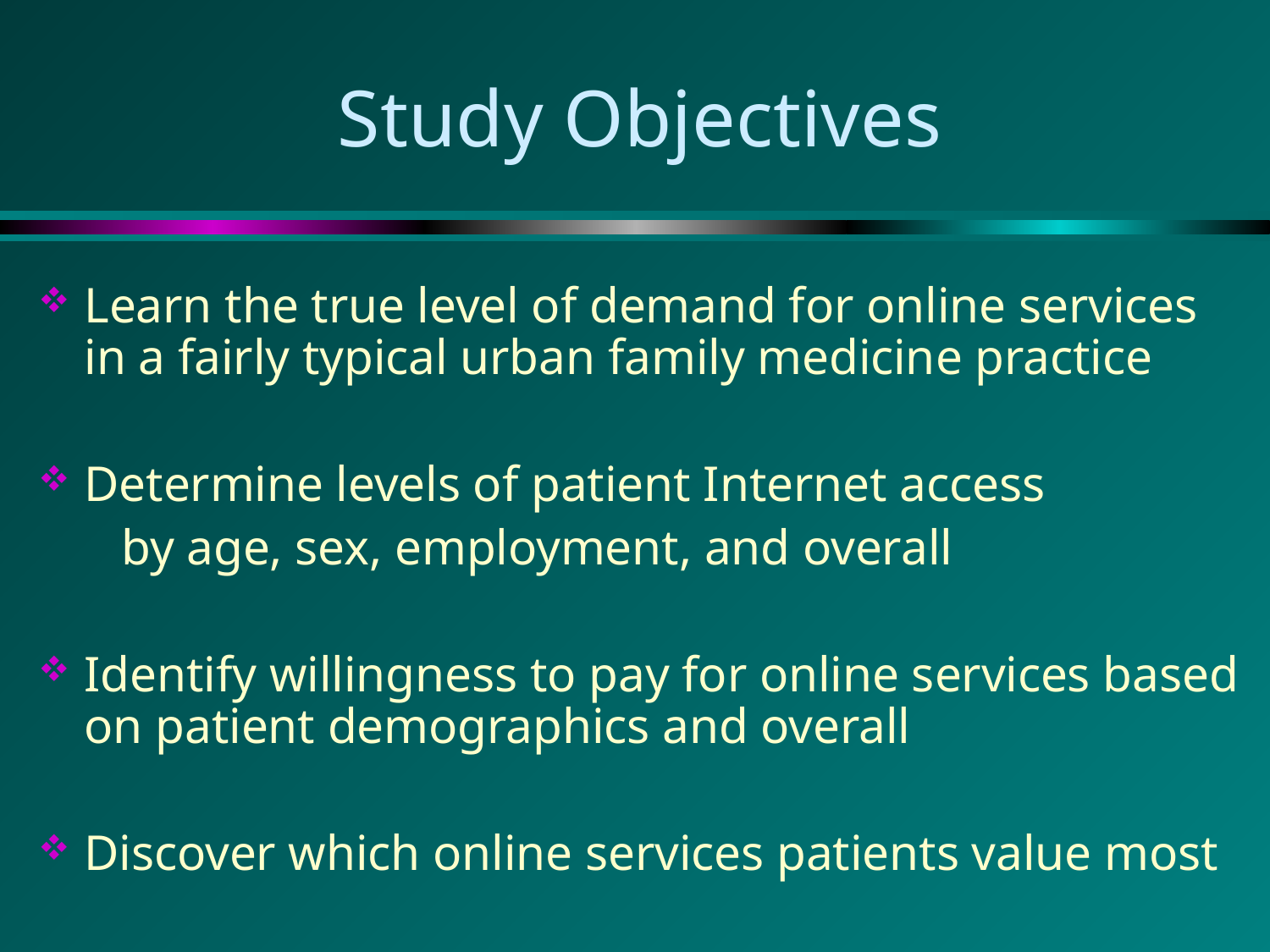

# Study Objectives
Learn the true level of demand for online services in a fairly typical urban family medicine practice
Determine levels of patient Internet access
 by age, sex, employment, and overall
Identify willingness to pay for online services based on patient demographics and overall
Discover which online services patients value most

## Slide 10
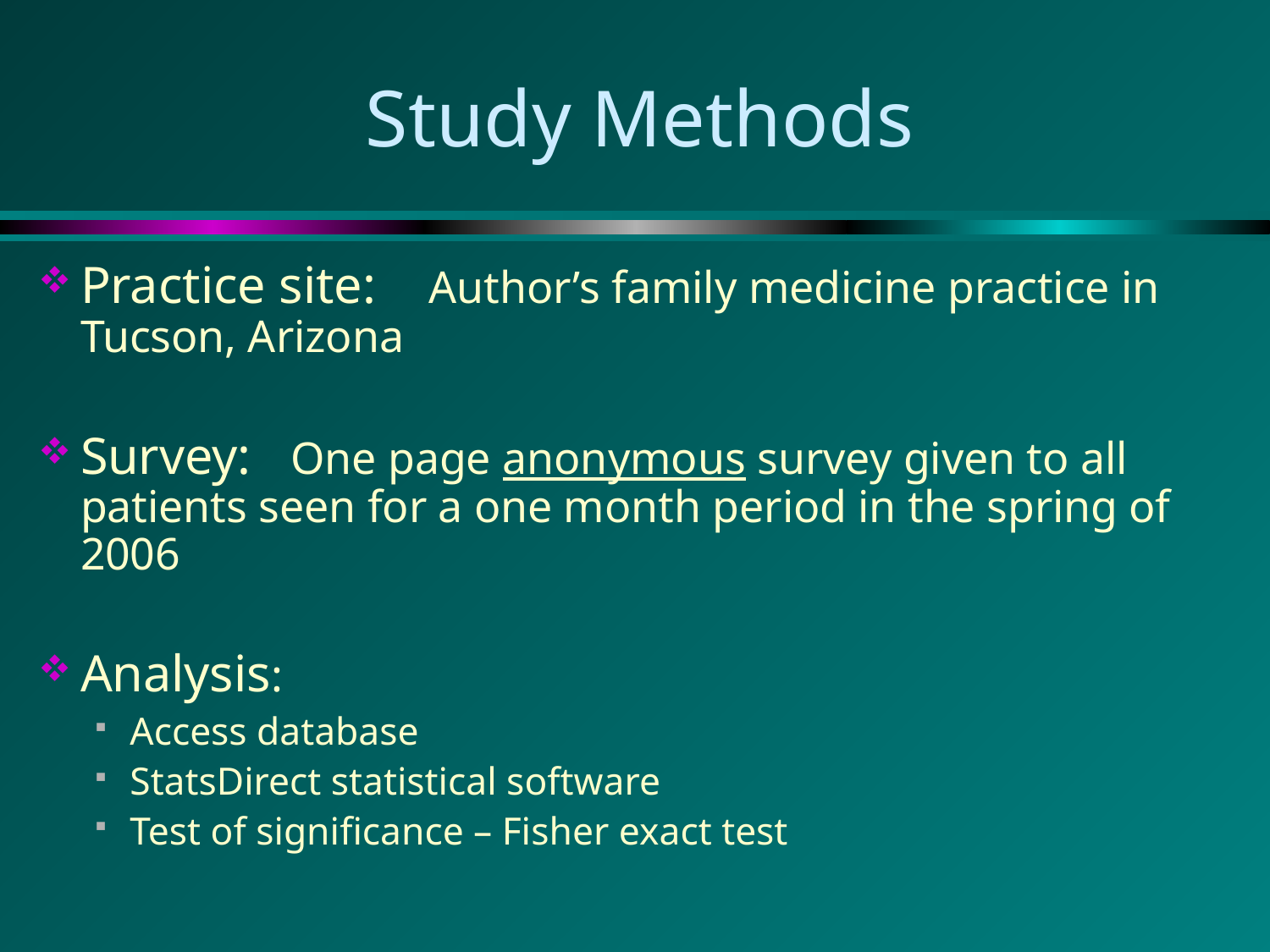

# Study Methods
Practice site: Author’s family medicine practice in Tucson, Arizona
Survey: One page anonymous survey given to all patients seen for a one month period in the spring of 2006
Analysis:
Access database
StatsDirect statistical software
Test of significance – Fisher exact test

## Slide 11
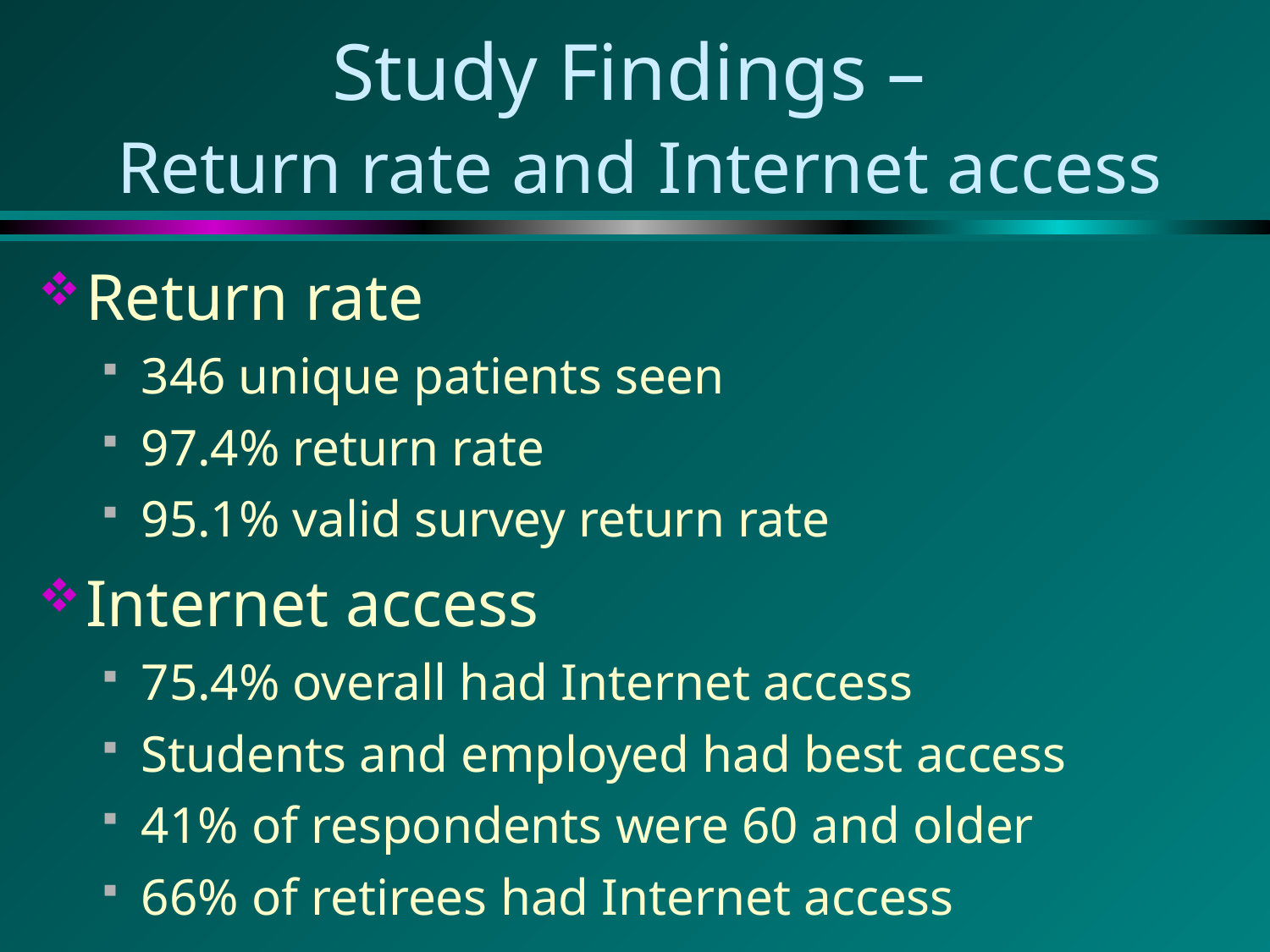

# Study Findings – Return rate and Internet access
Return rate
346 unique patients seen
97.4% return rate
95.1% valid survey return rate
Internet access
75.4% overall had Internet access
Students and employed had best access
41% of respondents were 60 and older
66% of retirees had Internet access

## Slide 12
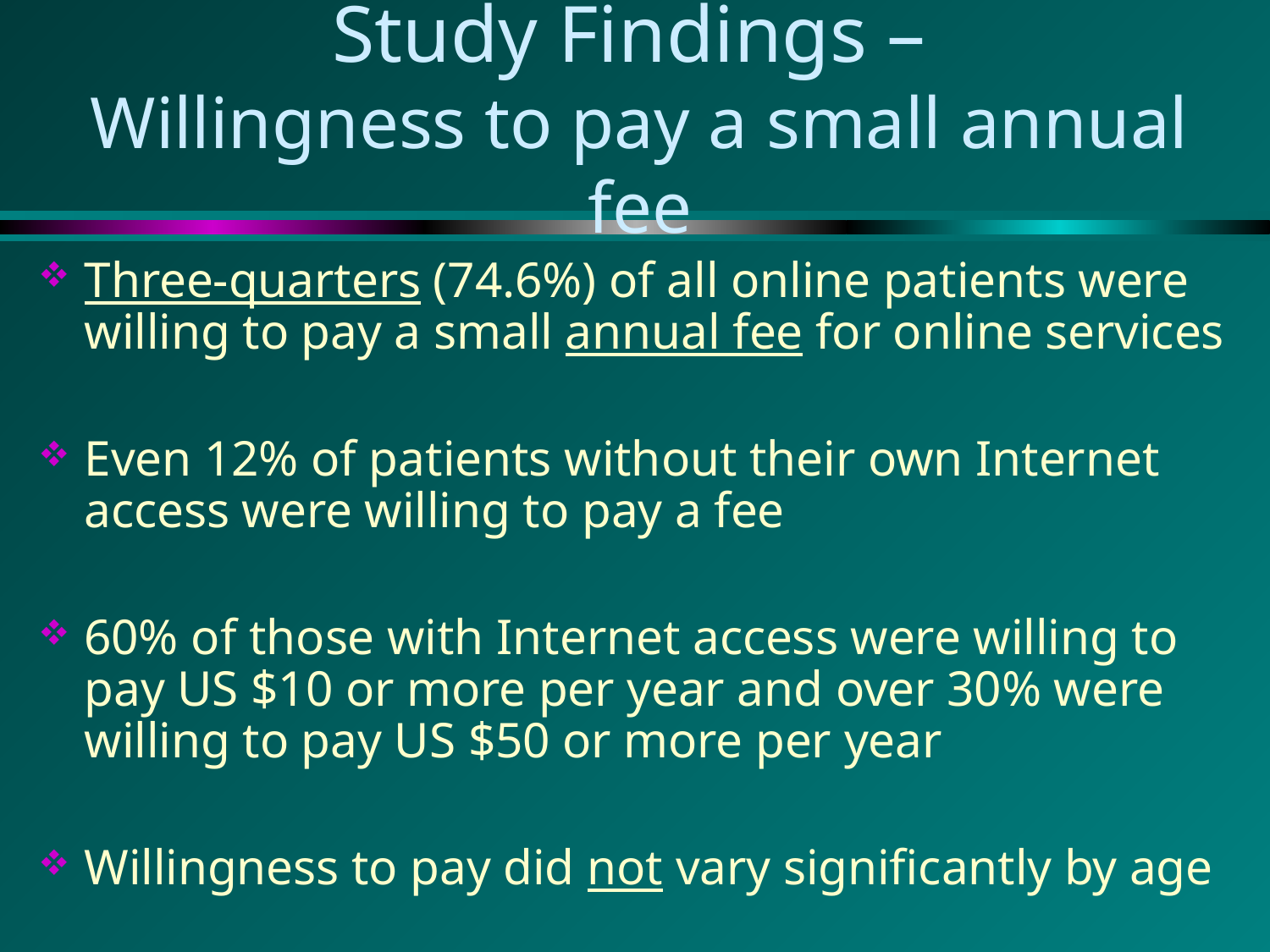

# Study Findings – Willingness to pay a small annual fee
Three-quarters (74.6%) of all online patients were willing to pay a small annual fee for online services
Even 12% of patients without their own Internet access were willing to pay a fee
60% of those with Internet access were willing to pay US $10 or more per year and over 30% were willing to pay US $50 or more per year
Willingness to pay did not vary significantly by age

## Slide 13
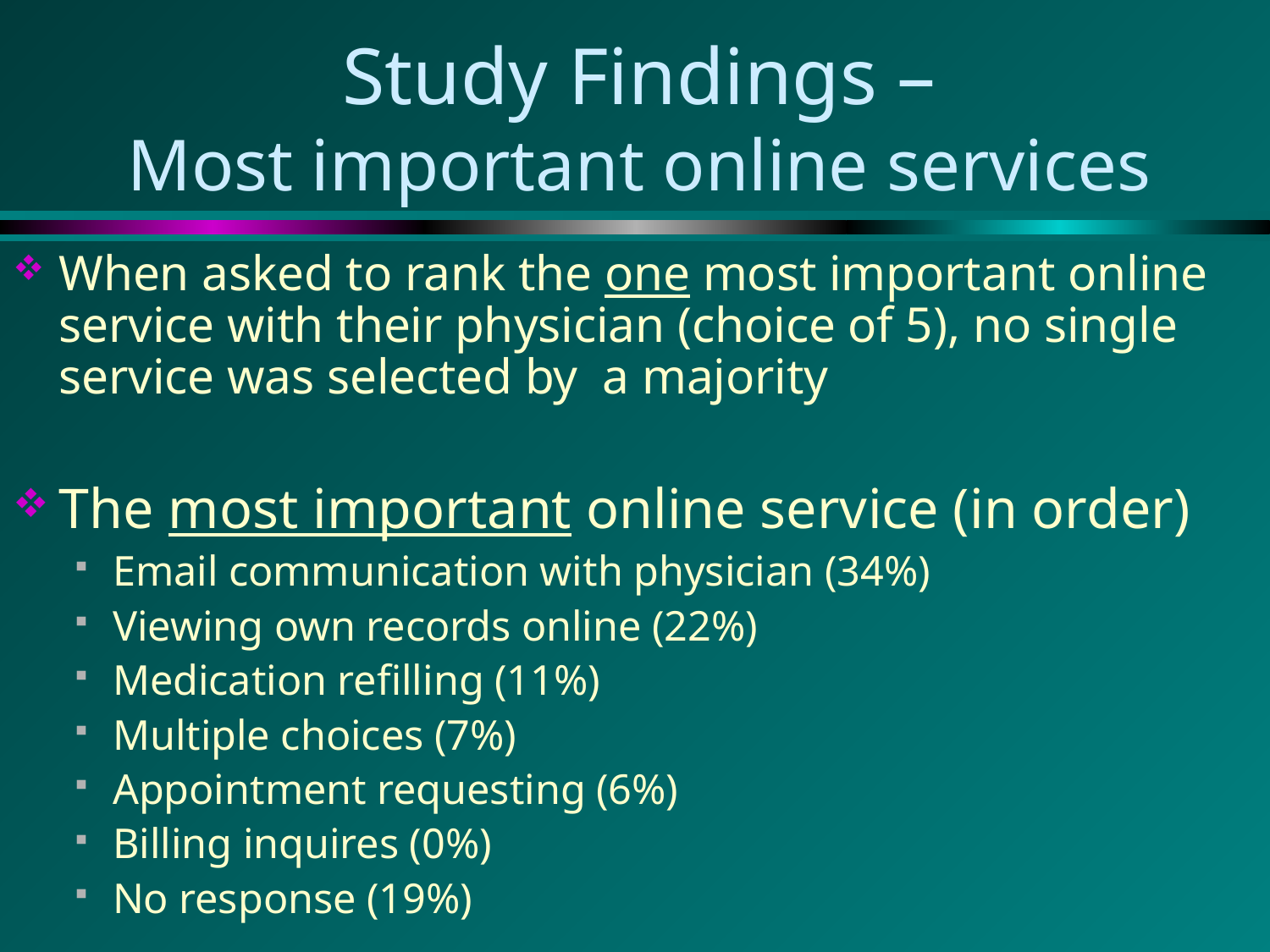

# Study Findings – Most important online services
When asked to rank the one most important online service with their physician (choice of 5), no single service was selected by a majority
The most important online service (in order)
Email communication with physician (34%)
Viewing own records online (22%)
Medication refilling (11%)
Multiple choices (7%)
Appointment requesting (6%)
Billing inquires (0%)
No response (19%)

## Slide 14
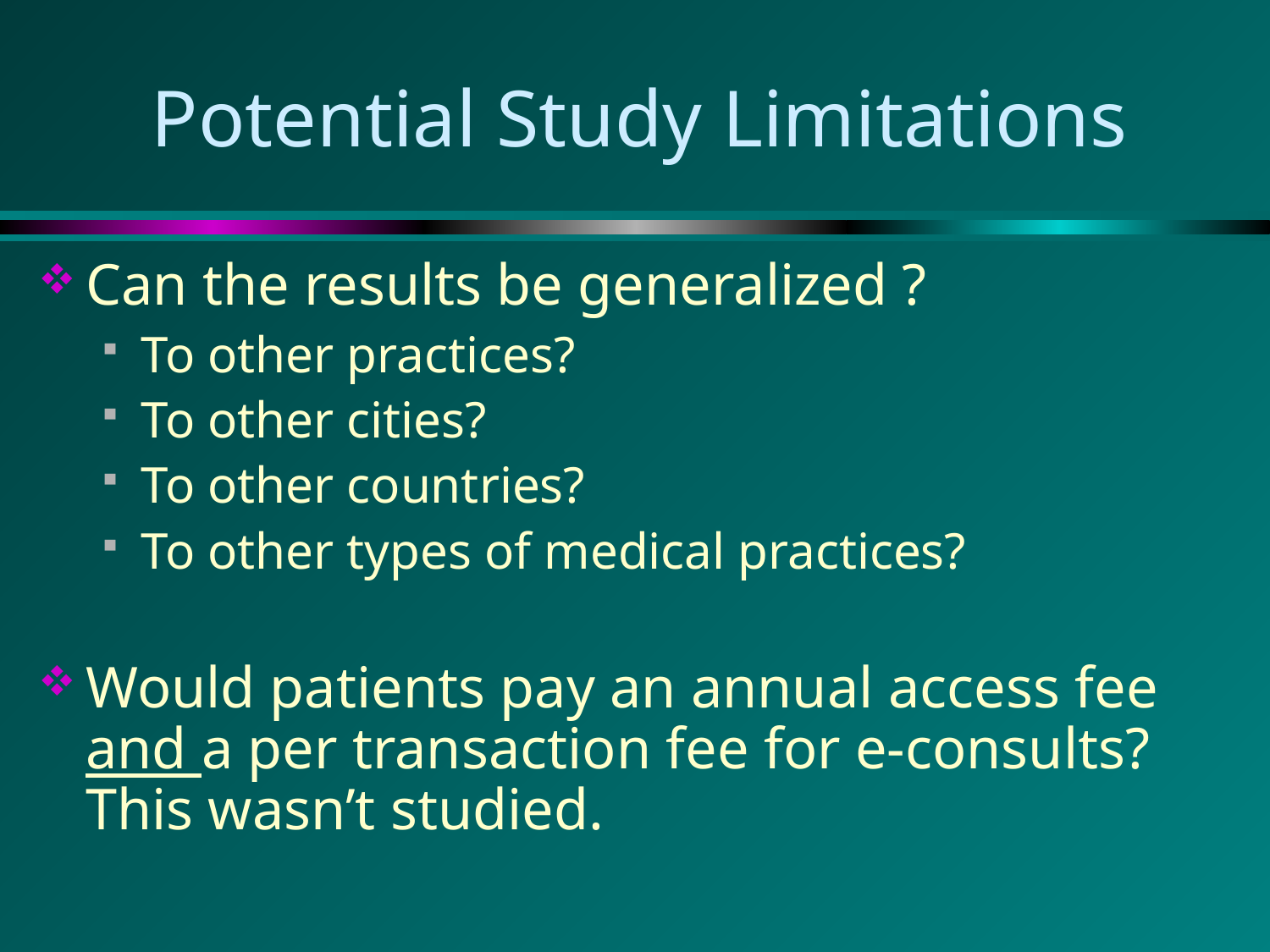

# Potential Study Limitations
Can the results be generalized ?
To other practices?
To other cities?
To other countries?
To other types of medical practices?
Would patients pay an annual access fee and a per transaction fee for e-consults? This wasn’t studied.

## Slide 15
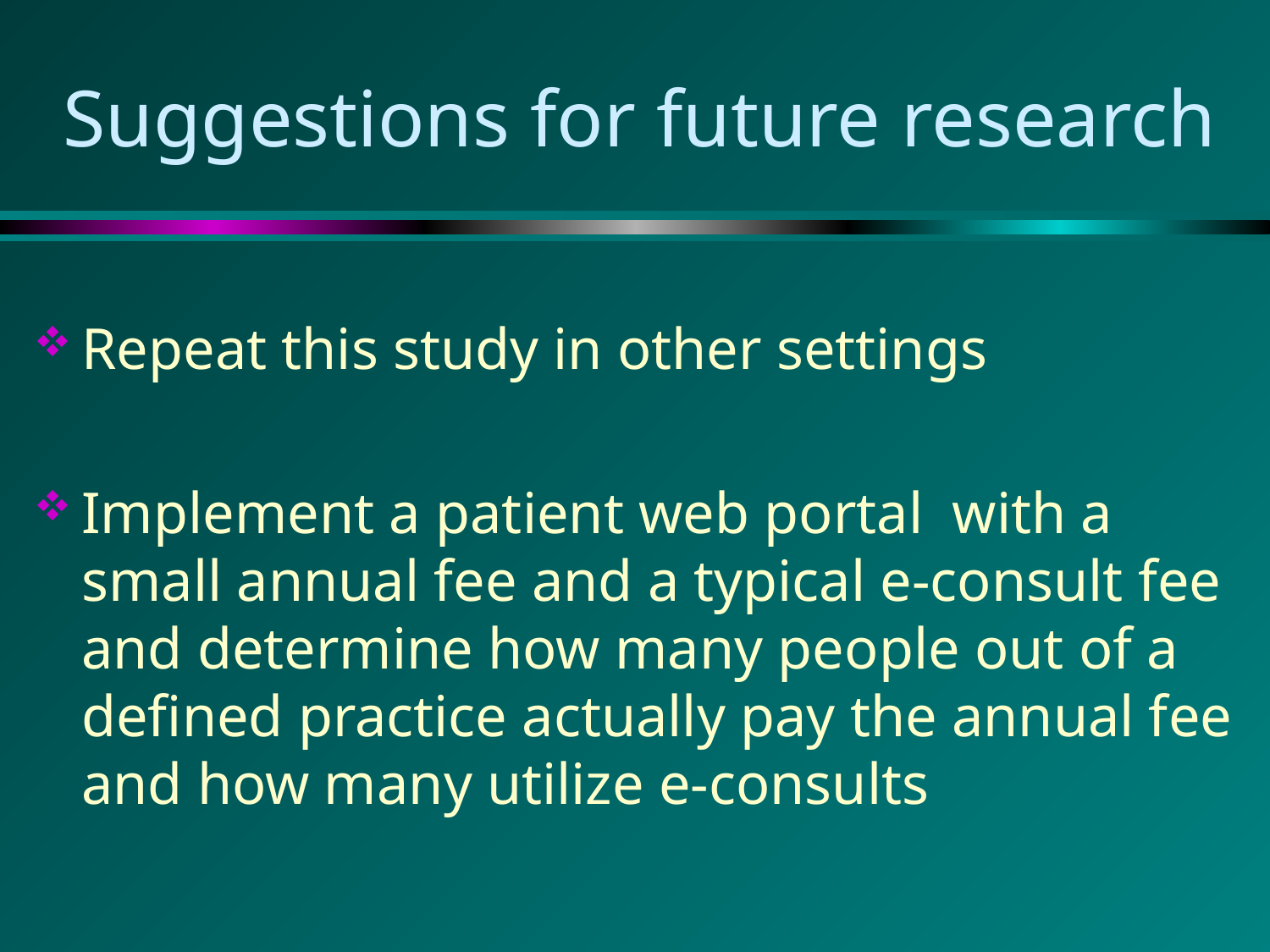

# Suggestions for future research
Repeat this study in other settings
Implement a patient web portal with a small annual fee and a typical e-consult fee and determine how many people out of a defined practice actually pay the annual fee and how many utilize e-consults

## Slide 16
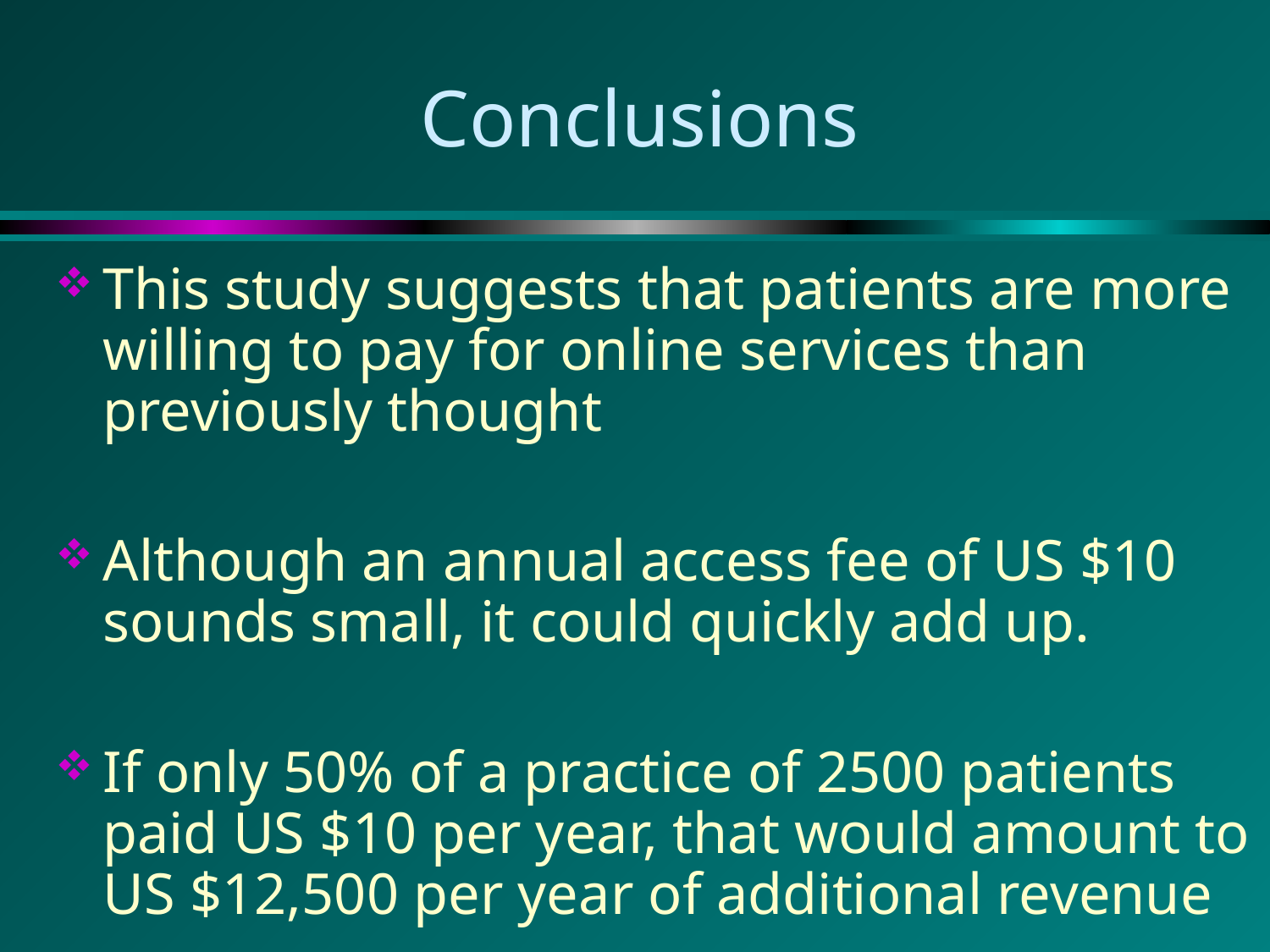

# Conclusions
This study suggests that patients are more willing to pay for online services than previously thought
Although an annual access fee of US $10 sounds small, it could quickly add up.
If only 50% of a practice of 2500 patients paid US $10 per year, that would amount to US $12,500 per year of additional revenue
